# Appropriate insertion point for percutaneous pedicle screw placement in the lumbar spine using c-arm fluoroscopy: a cadaveric study

**DOI:** 10.1186/s12891-020-03751-y

**Published:** 2020-11-14

**Authors:** Wei-Xing Xu, Wei-Guo Ding, Bin Xu, Tian-Hong Hu, Hong-Feng Sheng, Jia-Fu Zhu, Xiao-Long Zhu

**Affiliations:** Department of Orthopaedics, Tongde Hospital of Zhejiang Province, 234# Gu-cui Road, Hangzhou, 310012 People’s Republic of China

**Keywords:** C-arm X-ray fluoroscopy, Insertion point, Facet joint, Pedicle projection

## Abstract

**Background:**

We studied the characteristics and regularity of appropriate insertion points for percutaneous pedicle screw placement in the lumbar spine using C-arm X-ray fluoroscopy. The purpose of this study was to improve the accuracy of percutaneous pedicle screw placement and reduce the incidence of superior-level facet joint violation.

**Methods:**

Six normal spinal specimens were included. Three different methods for placing percutaneous pedicle screws in the lumbar spine were applied, including the Roy-Camille method, Magerl method and Weinstein method. The relationships among the insertion point, pedicle projection and proximal facet joint on C-arm X-ray films were studied. The projection morphology of the vertebral pedicle in different segments of the lumbar spine was observed. The relationship between the outer edge of the pedicle projection and the outer edge of the cranial articular process was also studied. The distance between the insertion point and the facet joint (M1), the distance between the insertion point and outer edge of the cranial articular process (M2), and the distance between the insertion point and the projection center of the pedicle (M) were measured.

**Results:**

In this study, we found that the projection shape of the vertebral pedicle differed across segments of the lumbar spine: the shape for L1-L3 was oval, and that for L4-L5 was round. The radiographic study showed that the outer edge of the cranial articular process was located on the lateral side of the outer edge of the pedicle projection and did not overlap with the pedicle projection. M for the Weinstein group was larger than that for the Roy-Camille group (*P* <  0.05). M1 for the Weinstein group was larger than that for the Roy-Camille and Magerl groups (*P* <  0.05). M2 for the Roy-Camille group was negative, M2 for the Magerl group was 0, and M2 for the Weinstein group was positive.

**Conclusion:**

Under C-arm X-ray fluoroscopy, we were able to accurately identify the characteristics and regularity of the appropriate insertion point for percutaneous pedicle screw placement in the lumbar spine, which was important for improving the accuracy of percutaneous pedicle screw placement and reducing the incidence of superior-level facet joint violation.

The rate of injury to articular process joints when percutaneous pedicle screws are used is higher than that when open-surgery screws are used, and injury to articular process joints mostly occurs in the lower lumbar vertebrae [1]. Although the use of three-dimensional computed topography (CT) navigation during surgery can effectively reduce the rate of injury to articular process joints, it has not yet been widely promoted in Chinese hospitals because of its high cost and the high-end technology required for navigation, and most hospitals still use two-dimensional C-arm X-ray machines for the positioning of percutaneous pedicle screws under fluoroscopy. Anatomical imaging studies of vertebral pedicles have demonstrated that from L1 to L5, the transverse inclination angle of the pedicle gradually increases, and the transverse diameter of the pedicle isthmus gradually widens [2–4]. The distance between the projection of the pedicle axis on the back of the corpus vertebrae and the ipsilateral articular process joint gradually increases. It was argued that the Weinstein method, when used for screw placement in the lower lumbar vertebrae (L3–S1), has a high success rate and little effect on the articular process joint at the end of the screw [5]. Yson et al. [6] argued that a more lateral insertion point for percutaneous screws placed in the lower lumbar vertebrae should be adopted during open surgery to avoid damaging the articular processes at the end of the screw. However, no relevant studies have reported the use of two-dimensional C-arm fluoroscopy for selecting the appropriate screw insertion points and reducing the rate of injury to the articular process joint at the end of the screw. Accordingly, additional research is required.

## Materials and methods

### Materials

In this study, six normal T12–S1 spine specimens from adults (provided by the Department of Anatomy at Zhejiang University) soaked in formaldehyde were selected. Their lateral and posterior soft tissue structures as well as the complete articular capsule structures of the lumbar articular process joint were preserved to maintain specimen continuity and integrity. The specimen pedicles were subjected to CT examinations so that the specimens with small pedicle structures (unsuitable for screw placement) could be excluded. In addition, specimens with dysplasia, metabolic metastatic diseases and degenerative disease were excluded.

### Methods

#### Three screw-insertion points

**Fig. 1:**
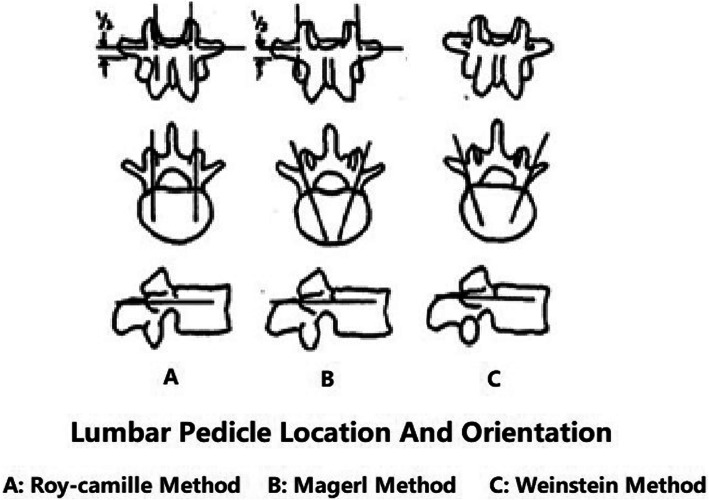
Characteristics and regularity of three different methods used for pedicle screw placement, including the Roy-Camille method, Magerl method and Weinstein method

For the Roy-Camille method, the insertion point is located at the intersection between the central axes of the cranial articular process and the transverse process. For the Magerl method, the screw is inserted at the intersection between the line perpendicular to the outer edge of the articular process and the central axis of the transverse process. For the Weinstein method, the insertion point is located at the intersection between the lower part of the outer edge of the cranial articular process and the central axis of the transverse process (Fig. 1). Under C-arm X-ray fluoroscopy guidance, specimens were drilled using a Kirschner wire from the insertion point along the pedicle axis to the vertebral body (the diameter of the Kirschner wire was 1.5 mm and the depth of drilling was also 1.5 mm), avoiding the articular process joint (maintaining more than 1 mm of distance from the articular process joint). The Kirschner wire was then removed from the insertion point. A 1.5-mm wire was then embedded at the insertion point to mark the three insertion points used for the three methods (Roy-Camille, Magerl, and Weinstein) of lumbar pedicle screw placement at the L1–L5 sections in the six lumbosacral spine specimens.

#### Imaging performance and measurement of screw insertion points

**Fig. 2:**
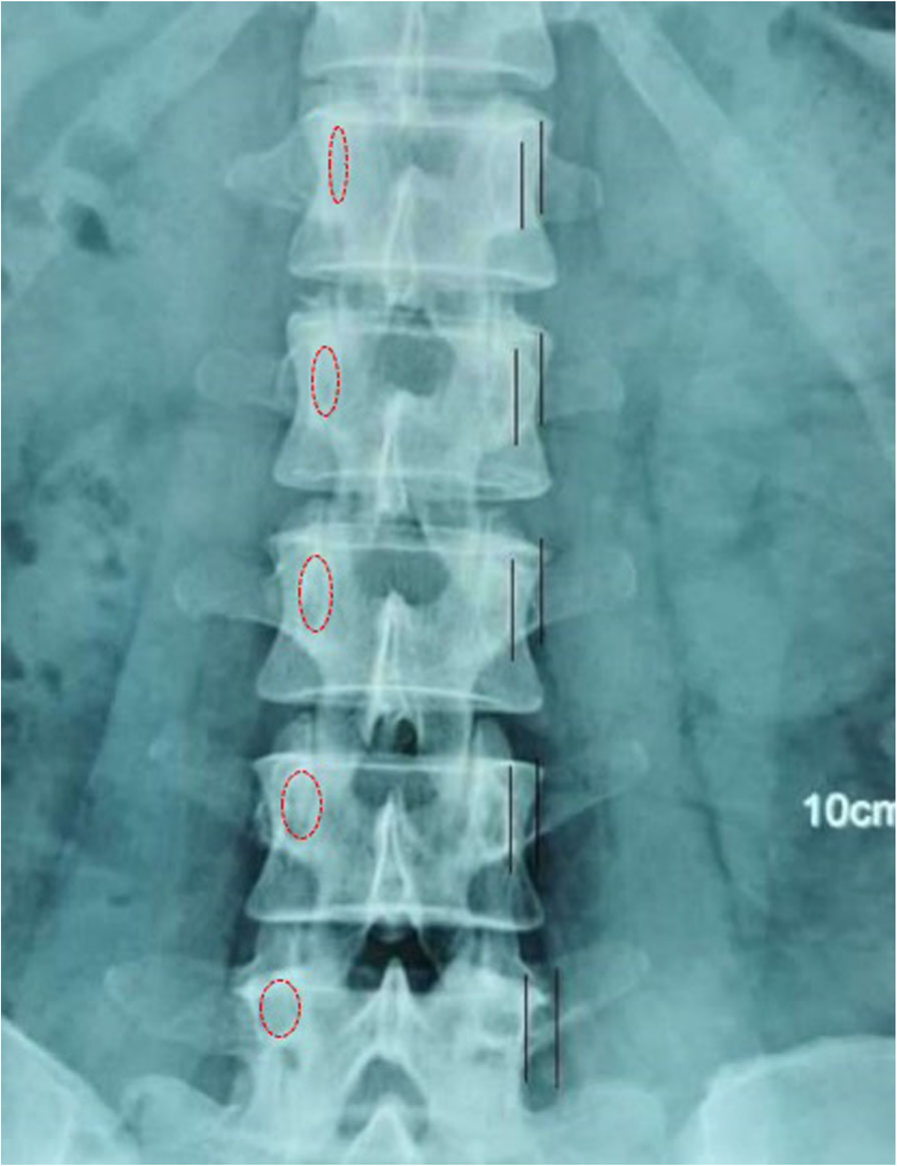
The projection shape of the vertebral pedicle in different segments of the lumbar spine. The outer edge of the cranial articular process was located on the lateral side of the outer edge of the pedicle projection and did not overlap with the pedicle projection. M1: the distances from different insertion points to the articular process joint, M2: the distances from different insertion points to the outer edge of the cranial articular process, M: the distances from different insertion points to the center of the pedicle projection

The insertion points of the specimens were fixed using metal markers. Then, standard anterior and posterior images of the specimens were taken with C-arm fluoroscopy (the standard anterior and posterior perspectives of the vertebral body were lined up with the upper and lower endplates, and the spinous process was aligned with the center of the pedicle on both sides). The C-arm fluoroscopic images were output in the DICOM format and input into an image processing system (Syngo Imaging, Siemens). All measurements were made in the image processing system. Then, the anatomical imaging relationships among the three common insertion points used in lumbar vertebrae pedicle screw placement, pedicle projection, and proximal articular process joints were assessed. The morphology of the pedicle projections of different sections of the lumbar vertebrae (L1–L5) as well as the relationship between the outer edge of the pedicle projection and that of the cranial articular process was assessed to determine whether the outer edge of the cranial articular process was located inside or outside of the outer edge of the pedicle projection (Fig. 2). In addition, the distances from different insertion points to the articular process joint (M1), to the outer edge of the cranial articular process (M2), and to the center of the pedicle projection (M) were recorded.

### Statistical analysis

SPSS 17.0 (SPSS, the United States) was used for statistical analysis. The measurement data (image distance) are expressed as the means ± standard deviations. One-way ANOVA was employed to compare the three groups, and *P* <  0.05 indicated statistical significance.

## Results

### Imaging of the pedicles and cranial articular processes

The projection shape of the vertebral pedicle differed across the segments of the lumbar spine: the shape for L1 - L3 was more oval, and that for L4 - L5 was more round (the aspect ratio was decreased from L1 to L5) (Table 1 and Fig. 2). The radiographic study showed that the outer edge of the cranial articular process was located on the lateral side of the outer edge of the pedicle projection and did not overlap with the pedicle projection (Fig. 2).

**Table 1:** Imaging of pedicles and cranial articular processes of lumber vertebral segments

Sections	Pedicle projection	Relationship between the outer edges of the pedicle projection and cranial articular process
L1	Elliptical	Lateral
L2	Elliptical	Lateral
L3	Elliptical	Lateral
L4	Round	Lateral
L5	Round	Lateral

### Measurements for the different methods

**Fig. 3:**
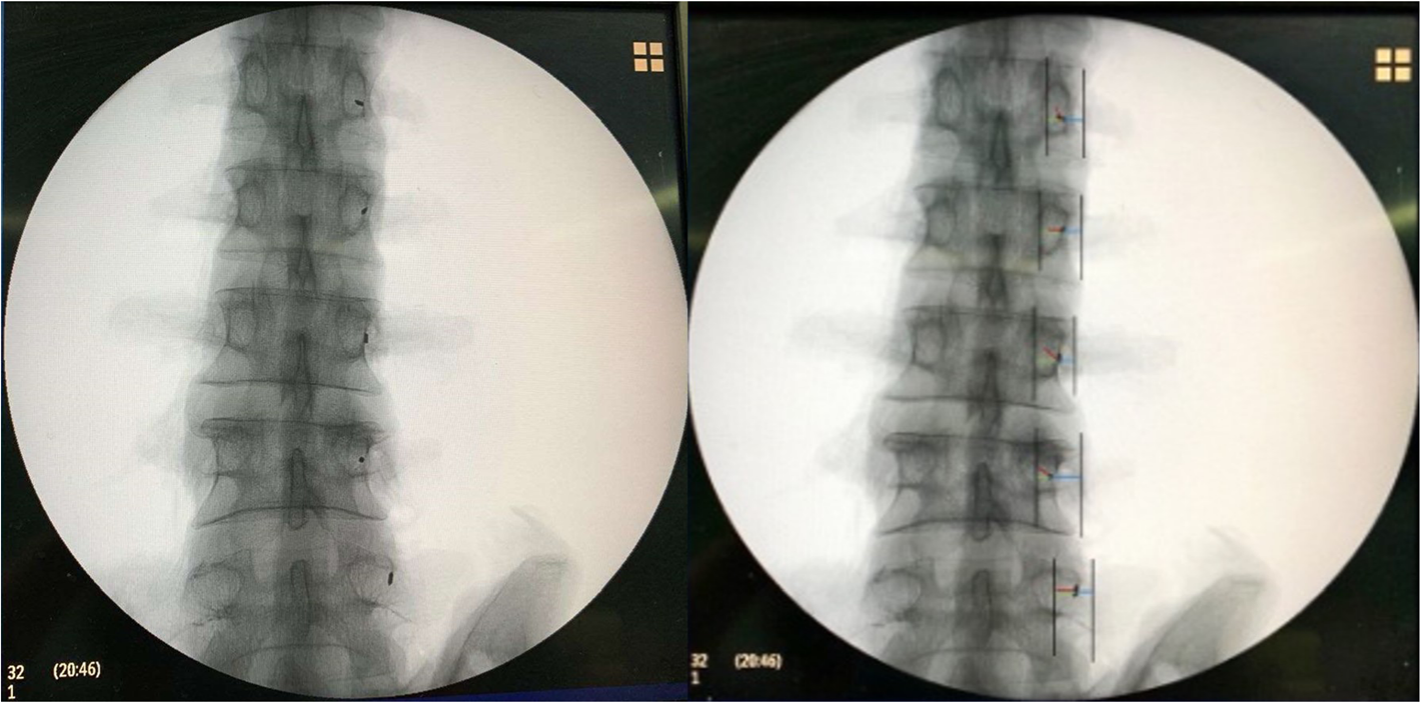
The appropriate insertion points used in the Roy-Camille group

**Fig. 4:**
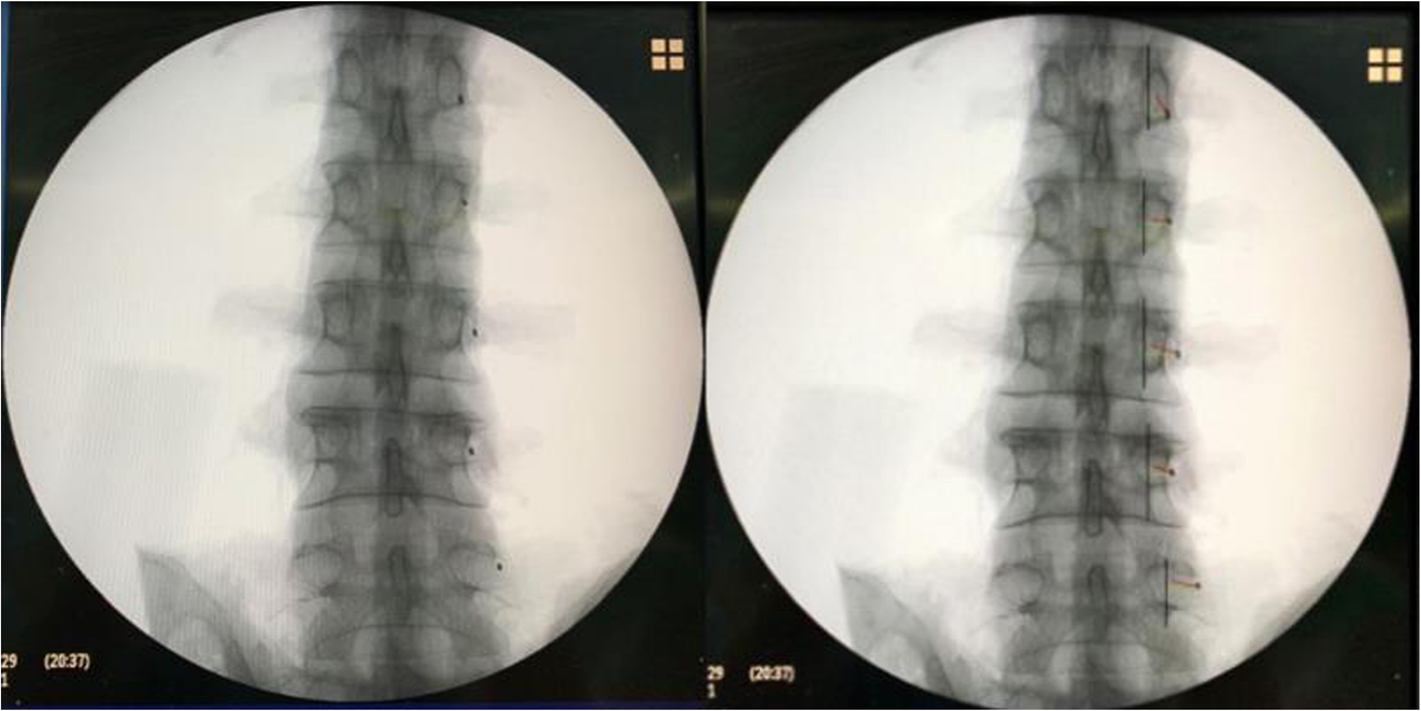
The appropriate insertion points used in the Magerl group

**Fig. 5:**
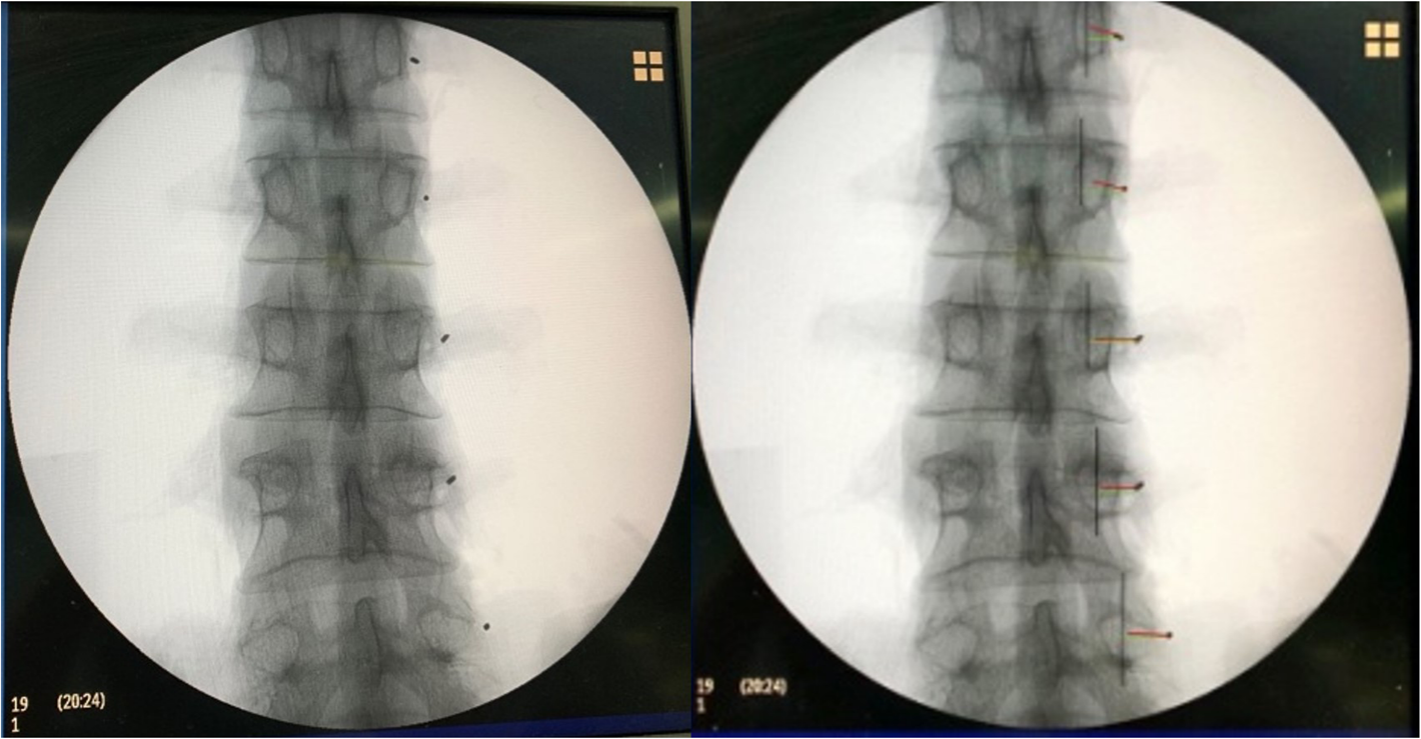
The appropriate insertion points used in the Weinstein group

In L1 and L2, M was similar among the three groups (P1 = 0.158; P2 = 0.086). In L3, L4 and L5, the difference in M among the three groups was statistically significant (P3 = 0.025, P4 = 0.034, P5 = 0.037). The differences in M between the Roy-Camille and Magerl groups and between the Magerl and Weinstein groups were not statistically significant. M for the Weinstein group was larger than that for the Roy-Camille group, and the difference was statistically significant (*P* <  0.05) (Table 2). In L1, M1 was similar among the three groups (P1 = 0.077). In L2, L3, L4 and L5, the difference in M1 among the three groups was statistically significant (P2 = 0.015, P3 = 0.018, P4 = 0.021, P5 = 0.030). The difference in M1 between the Roy-Camille and Magerl groups was not statistically significant. M1 for the Weinstein group was larger than that for the Roy-Camille and Magerl groups, and the difference was statistically significant (*P* <  0.05) (Table 3). M2 for the Roy-Camille group was negative (the insertion point was located on the inner side of the outer edge of the cranial articular process), M2 for the Magerl group was 0 (the insertion point was located at the outer edge of the cranial articular process), and M2 for the Weinstein group was positive (the insertion point was located on the outer side of the outer edge of the cranial articular process) (Table 4). The appropriate insertion points for the Roy-Camille, Magerl and Weinstein groups are shown in Figs. 3, 4, and 5, respectively.

**Table 2:** The comparison of M of different methods (x ± s, *n* = 12)

Sections	Roy–Camille	Magerl	Weinstein	*P*-value
L1	1.5 ± 0.5	1.6 ± 0.3	1.8 ± 0.3	0.158
L2	1.8 ± 0.5	2.0 ± 0.5	2.3 ± 0.6	0.086
L3	2.3 ± 0.8	2.8 ± 0.7	3.2 ± 0.8^γ^	0.025
L4	3.1 ± 0.9	3.7 ± 1.1	4.3 ± 1.2^γ^	0.034
L5	4.1 ± 1.1	4.7 ± 1.3	5.5 ± 1.4^γ^	0.037

γ: comparison between Roy–Camille and Weinstein (*p <*  0.05)

**Table 3:** The comparison of M1 of different methods (x ± s, *n =* 12)

Sections	Roy–Camille	Magerl	Weinstein	*P-*value
L1	2.0 ± 0.6	2.0 ± 0.6	2.5 ± 0.6	0.077
L2	2.3 ± 0.6	2.4 ± 0.6	3.0 ± 0.6^βγ^	0.015
L3	2.8 ± 0.7	3.0 ± 0.8	3.7 ± 0.8^γ^	0.018
L4	4.1 ± 0.8	4.4 ± 0.9	5.2 ± 1.1^γ^	0.021
L5	5.2 ± 1.1	5.6 ± 1.2	6.5 ± 1.2^γ^	0.030

β: comparison between Magerl and Weinstein (*p <* 0.05); γ: comparison between Roy–Camille and Weinstein (*p <* 0.05)

**Table 4:** The comparison of M2 of different methods (x ± s, *n =* 12)

Sections	Roy–Camille	Magerl	Weinstein	*P-*value
L1	−1.2 ± 0.3	0^α^	1.5 ± 0.5^βγ^	< 0.001
L2	−1.5 ± 0.3	0^α^	1.9 ± 0.6^βγ^	< 0.001
L3	−2.0 ± 0.8	0^α^	2.1 ± 0.7^βγ^	< 0.001
L4	−3.2 ± 1.1	0^α^	2.5 ± 0.8^βγ^	< 0.001
L5	−4.3 ± 1.7	0^α^	2.8 ± 0.9^βγ^	< 0.001

α: comparison between Roy–Camille and Magerl (*p <* 0.05); β: comparison between Magerl and Weinstein (*p <* 0.05); γ: comparison between Roy–Camille and Weinstein (*p <* 0.05)

## Discussion

Since the rapid development of minimally invasive spinal surgery, percutaneous pedicle screw fixation has gradually become known as a common, basic technique [7]. Compared with conventional screw placement through open surgery, percutaneous pedicle screw fixation does not require ablation of the erector spinae, leads to less blood loss, and leads to less severe lower-back pain, while accelerating recovery after surgery [8]. However, percutaneous screw placement requires the aid of imaging; selecting the optimal insertion point on the basis of naked-eye observations of anatomical landmarks is impossible. Because the optimal insertion point cannot be selected on the basis of naked-eye observations, screw placement without imaging guidance is likely to lead to cranial articular process joint injuries [9]. Patel et al. performed pedicle screw placement from L1 to S1 by using C-arm fluoroscopy, and afterwards, the cadaver was dissected, and its articular process joints were exposed [10]. The authors calculated a 58% injury rate to the cranial articular process joints after removing the screws. Park et al. assessed the postoperative damage to the cranial articular process joints caused by percutaneous pedicle screws and reported a 31.5% injury rate (58/184) [1]. The injury rate reported by Knox et al. was only 6.6% (8/122) for injuries to the cranial articular process joints that occurred during percutaneous pedicle screw fixation [11]. In addition, the rate of injury to cranial articular process joints is affected by the insertion point. The insertion point used in the Magerl method is located at the intersection between the line perpendicular to the lateral edge of the articular process and the central axis of the transverse process, whereas that used in the Roy-Camille method is located at the intersection between the central axes of the cranial articular process and the transverse process. The Weinstein method in particular prevents damage to the cranial articular process joints, as pedicle screws are inserted at the lower lateral part of the cranial articular process. In a cadaver experiment, Chung et al. compared the effects of different insertion points on cranial articular process joints and demonstrated that using the mamillary process (the bony bulge above the articular process of the lumbar vertebrae) yielded an 8% higher rate of injury to cranial articular process joints than did the insertion point used in the Magerl method [12]. In a clinical review and analysis conducted by Chen et al., the rate of injury to the cranial articular process was as high as 100% with the Roy-Camille method, whereas that with the Weinstein method was only 23.8%. Improvements in image navigation and computer-aided navigation technology in spinal surgery have enhanced the accuracy of pedicle screw fixation [13]. With the use of three-dimensional CT navigation during percutaneous pedicle screw fixation, clinicians can simulate insertion points and path directions in real time. Compared with conventional two-dimensional C-arm fluoroscopy, three-dimensional CT can lead to large improvements in the accuracy and safety of pedicle screw placement, as well as reduce the time required for screw placement and degree of radiation exposure [14–16]. Using three-dimensional CT navigation during surgery can also reduce the occurrence of percutaneous pedicle screw injuries to the cranial articular process joints [17, 18]. However, because of the high-end technology required for navigation and high cost of instruments, this method has not been widely promoted in domestic hospitals. Most hospitals still use two-dimensional C-arm fluoroscopy for percutaneous pedicle screw placement.

Percutaneous screw placement was initially performed using pedicle coaxial fluoroscopy, and the center of the elliptical pedicle projection was used as the insertion point. This method of screw placement is cumbersome because adjusting the X-ray projection to the common axis of the pedicle is difficult. Because clinicians now have an in-depth understanding of pedicle imaging anatomy, standard lateral fluoroscopy of the vertebral body for screw placement is gradually being performed more frequently [19]. The shape of the pedicle projection under standard lateral fluoroscopy is an ellipse, and the anatomical axis of the pedicle is located on the outer edge of the center of the elliptical projection. The three insertion points used in the three percutaneous screw placement methods (Roy-Camille, Magerl, and Weinstein methods) at the L1–L5 sections of the lumbar vertebrae yield different probabilities of injury to the cranial articular process joints. The Weinstein method produces the fewest injuries, followed by the Magerl method, and the Roy-Camille method yields the most injuries. Therefore, accurately locating these insertion points by using C-arm fluoroscopy is critical. This method requires that the laws of anatomical imaging are explored to identify appropriate insertion points, improve the quality of percutaneous screw placement, and reduce the occurrence of injuries to cranial articular process joints, thereby reducing the incidence of spondylosis. This study demonstrated that different sections of the lumbar vertebrae have different pedicle projections; L1–L3 exhibited elliptical projections, and L4 and L5 displayed round projections, the aspect ratio was decreased from L1 to L5. This finding means that the pedicles in the upper lumbar vertebrae are tall and narrow, and the pedicle width gradually increases toward the lower lumbar vertebrae. The outer edge of the pedicle projection did not overlap with that of the cranial articular process. Fluoroscopy imaging studies have indicated that the outer edge of the cranial articular process is located at the outer edge of the pedicle projection (Fig. 3). The insertion point used in the Roy-Camille method is located at the 3 o’ clock and 4 o’ clock positions on the outer edge of the pedicle projection. Thus, this point has the shortest distance to the articular process joint (M_1_); moreover, its distance to the outer edge of the cranial articular process (M_2_) is negative. The distance to the center of the pedicle projection (M) is also the shortest for this point; however, the distance gradually increases from L1 to L5. The insertion point used in the Magerl method is located at the intersection between the outer edge of the projection of the cranial articular process and the transverse process. Its distance to the articular process joint (M_1_) is larger than that of the insertion point used in the Roy-Camille method; however, its distance to the outer edge of the cranial articular process (M_2_) is 0, indicating that the insertion point is on the outer edge of the cranial articular process. Moreover, the distance from the insertion point to the center of the pedicle projection (M) in the Magerl method is greater than that in the Roy-Camille method, and it gradually increases from L1 to L5. The insertion point used in the Weinstein method is located at the base of the transverse process bisector, which is located on the lower part of the outer edge of the cranial articular process. Its distance to the articular process joint (M_1_) is the largest, and its distance to the outer edge of the cranial articular process (M_2_) is positive, indicating that the insertion point is located at the outer edge of the cranial articular process. The distance from the insertion point to the center of the pedicle projection (M) is the largest, but it also gradually increases from L1 to L5.

There were several limitations of this study: (1) the size of spine specimens was relatively small, a bigger sample size would provide stronger evidence; (2) we didn’t consider the influence of height and weight of the specimens, different specimens with different sizes, the measurements may be different.

This study involved imaging research and proposed that the rate of injury to the articular process joint varies by the insertion point used and the section of the lumbar vertebrae in which the percutaneous screws are inserted. In addition, this study revealed that percutaneous pedicle screw placement in the lower lumbar vertebrae is likely to cause severe injury to articular process joints. The Roy-Camille method yielded the largest damage to articular process joints, followed by the Magerl method, whereas the Weinstein method caused the least damage. This study demonstrated that the appropriate insertion points for percutaneous screw placement can be located accurately with C-arm fluoroscopy and that the insertion points used in the Weinstein method can reduce the rate of injury to the articular process joint, thereby reducing the occurrence of spondylosis.

## Data Availability

The datasets used and/or analysed during the current study are available from the corresponding author on reasonable request.

## Abbreviation

CT: Computed topography
